# Nitrate of Silver as a Uterine Caustic

**Published:** 1878-12

**Authors:** 


NITRATE OF SILVER AS A UTERINE CAUSTIC, is thus spoken of by Dr. T. Addis Emmet: "We have no remedy which acts with more promptness than the nitrate of silver, which applied to the mucous membrane of the cervix, yet it has done more damage than any other. From being in common use it is the more dangerous; for its repeated action will ultimately destroy the mucous follicles, harden the tissues, and close the os, as certain as the application of the actual cautery."—*Hospital Gazette.*

